# Reactivation of latent tuberculosis through modulation of resuscitation promoting factors by diabetes

**DOI:** 10.1038/s41598-021-99257-1

**Published:** 2021-10-05

**Authors:** Arpana Verma, Maninder Kaur, Lakshya Veer Singh, Divya Aggarwal, Indu Verma, Bishan D. Radotra, Sadhna Sharma

**Affiliations:** 1 Department of Biochemistry, Postgraduate Institute of Medical Education and Research, Chandigarh, India; 2 TACF, International Centre for Genetic Engineering and Biotechnology, New Delhi, India; 3 Department of Histopathology, Postgraduate Institute of Medical Education and Research, Chandigarh, India

**Keywords:** Microbiology, Molecular biology, Diseases, Pathogenesis, Risk factors

## Abstract

The evidence of an association between diabetes and latent tuberculosis infection (LTBI) remains limited and inconsistent. Thus, the study aims to delineate the role of diabetes in activation of latent tuberculosis infection. Murine model of latent tuberculosis and diabetes was developed, bacillary load and gene expression of resuscitation promoting factors (*rpfA-E*) along with histopathological changes in the lungs and spleen were studied. Treatment for LTBI [Rifampicin (RIF) + Isoniazid (INH)] was also given to latently infected mice with or without diabetes for 4 weeks. Diabetes was found to activate latent tuberculosis as the colony forming unit (CFU) counts were observed to be > 10^4^ in lungs and spleen. The gene expression of *hspX* was downregulated and that of *rpfB* and *rpfD* was observed to be upregulated in latently infected mice with diabetes compared to those without diabetes. However, no significant reduction in the CFU counts was observed after 4 weeks of treatment with RIF and INH. Diabetes helps in the progression of LTBI to active disease mainly through altered expression of resuscitation promoting factors *rpfB* and *rpfD*, which can serve as important targets to reduce the shared burden of tuberculosis and diabetes.

## Introduction

*Mycobacterium tuberculosis* infects nearly 25% of the world’s population out of which only 5–10% develop active disease while 90–95% people live with clinically asymptomatic latent tuberculosis infection (LTBI). This accounts for a huge reservoir of LTBI infected individuals^1^. Latent form of *M. tuberculosis* has slow replication rates and is metabolically less active relative to its active form^2,3^. *M. tuberculosis* is able to survive in oxygen deprived environment through altered gene expression, modulated metabolic pathways and switch over to anaerobic respiration^4–10^. All these adaptations contribute to the development of LTBI in humans. However, the compromised state of immune system as in HIV, undernutrition, smoking and diabetes, imbalances this equilibrium leading to reactivation of LTBI and progression to active disease.

Diabetes mellitus (DM) is among the top three common risk factor for tuberculosis along with HIV and undernutrition^11^. Diabetic patients are two times more susceptible to develop TB as compared to non-diabetics and this alliance is more substantial in HIV-positive patients^12^. Diabetes increases early mortality risk among TB patients, irrespective of their HIV status^13^. Presently, the exact mechanisms behind tuberculosis and diabetes association are unclear. Exogenous factors primarily control the acquisition of infection whereas immune insufficiency plays a pivotal role in the reactivation of disease^14^. TB diabetes has become an impending co-epidemic and reasons for this could be rise in diabetes and unawareness of people about its risk, huge reservoir of LTBI and active tuberculosis^15^.

Studies on growth of *M. tuberculosis* cultures under hypoxia combined with genetic analysis have illustrated an amplified expression of a 16 kDa α-crystallin homolog protein. Apart from this, resuscitation promoting factors (RPFs) of *M. tuberculosis* have been shown to stimulate the growth of dormant mycobacteria. *Mycobacterium tuberculosis* consists of five *rpf* genes i.e. *rpfA* (Rv0867c), *rpfB* (Rv1009), *rpfC* (Rv1884c), *rpfD* (Rv2389c) and *rpfE* (Rv2450c) with lysozyme activity that aid in reactivation via hydrolysis of the peptidoglycans^16,17^. Since diabetes is known to play an important role in activating latent tuberculosis, it is necessary to study the role of various *rpf* genes and other associated genes in order to elucidate the mechanism of activation of latent tuberculosis in diabetes.

Therefore, an animal model of latent tuberculosis and diabetes was used to study the effect of diabetes on latent tuberculosis activation and its treatment outcome.

## Results

### Diabetes increased the bacillary load in latent TB mice

**Figure 1:**
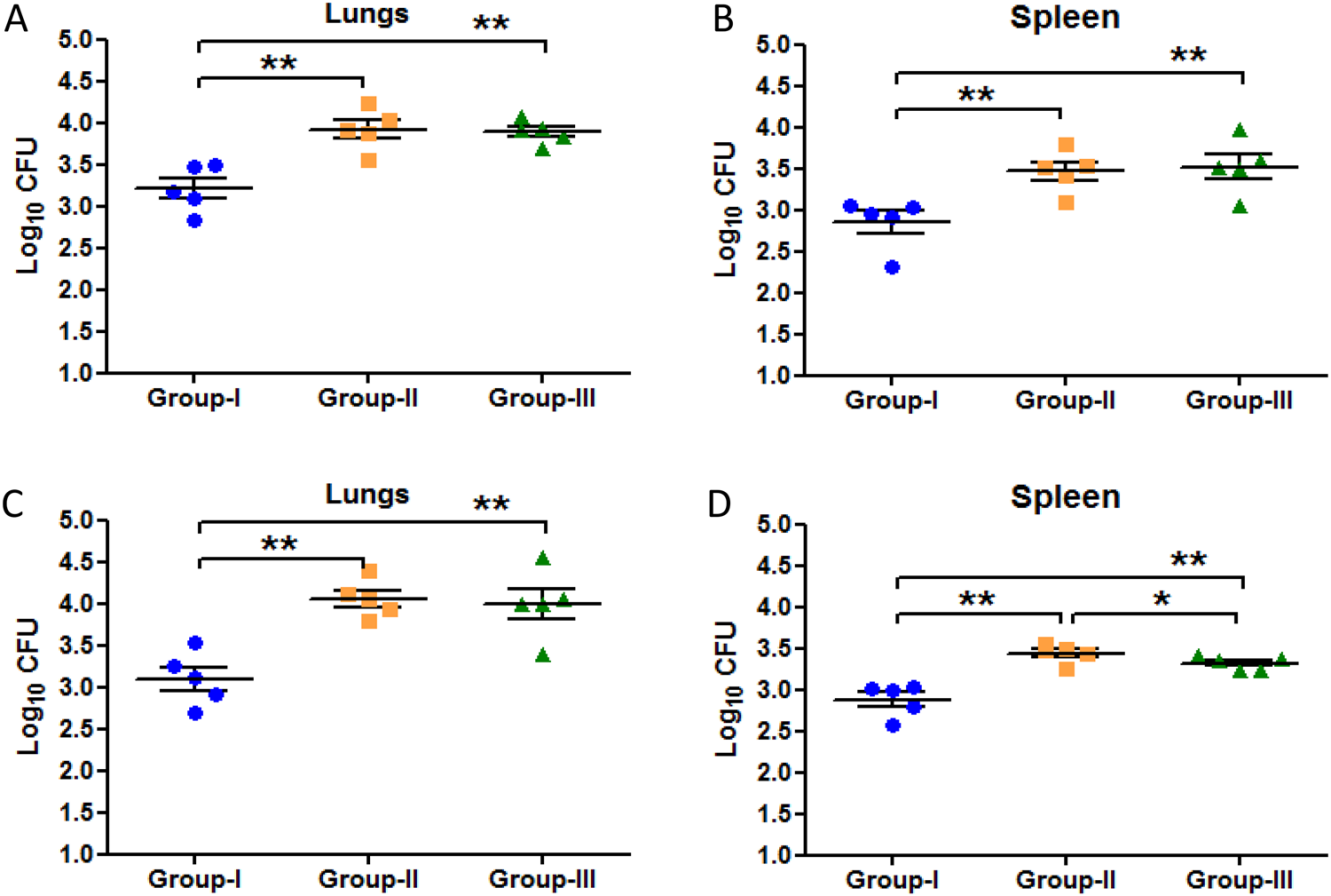
CFU enumeration of *M. tuberculosis* H37Rv in latent tuberculosis after diabetes development. Log_10_ CFU counts in lungs and spleen of mice from Group-I, Group-II and Group-III at different time points of streptozotocin treatment. (**A**) Lungs after 9 weeks (**B**) Spleen after 9 weeks (**C**) Lungs after 13 weeks (**D**) Spleen after 13 weeks of streptozotocin treatment. The data were represented as Mean ± SE of 5 animals in each group and the statistical significance was determined by Mann–Whitney test. **p ≤ 0.01, *p ≤ 0.05. Group-I: latent tuberculosis only, Group-II: latent tuberculosis with diabetes and Group-III: latent tuberculosis with immunosuppression. Animal model was developed only once but CFU enumeration was done in triplicates from each animal.

Murine model of latent tuberculosis was successfully developed as the CFU counts were < 10^4^ in lungs and spleen along with the formation of granuloma in lungs of latent TB group after 4 and 6 weeks of *M. tuberculosis* infection. After the establishment of latent tuberculosis, animals were divided in three groups i.e. Group-I (latent tuberculosis only), Group-II (latent tuberculosis with diabetes) and Group-III (latent tuberculosis with immunosuppression). Diabetes was induced in Group-II animals and was established at week 8 as blood glucose levels were found to be higher than 200 mg/dl. Two weeks after development of diabetes i.e. week 9, the mean CFU counts in lungs and spleen of Group-II and Group-III were found to be significantly increased as compared to Group-I (Fig. 1A,B). However, no significant difference was observed between the mean CFU counts in lungs and spleen of Group-II and Group-III animals. After 6 weeks of diabetes development, i.e. week 13, the mean CFU counts in lungs and spleen of Group-I animals were found to be 3.4 ± 0.14 log_10_ and 3.18 ± 0.09 log_10_ respectively, which signifies the maintenance of latency. Moreover, approximately one log increase in mean CFU counts was observed in the lungs and more than one log increase was observed in spleen of Group-II animals as compared to Group-I animals indicating the activation of latent tuberculosis infection in Group-II. Likewise, in Group-III approximately one log increase in mean CFUs of lungs and spleen was observed as compared to Group-I (Fig. 1C,D).

### Diabetes affected the granuloma formation in the lungs

**Figure 2:**
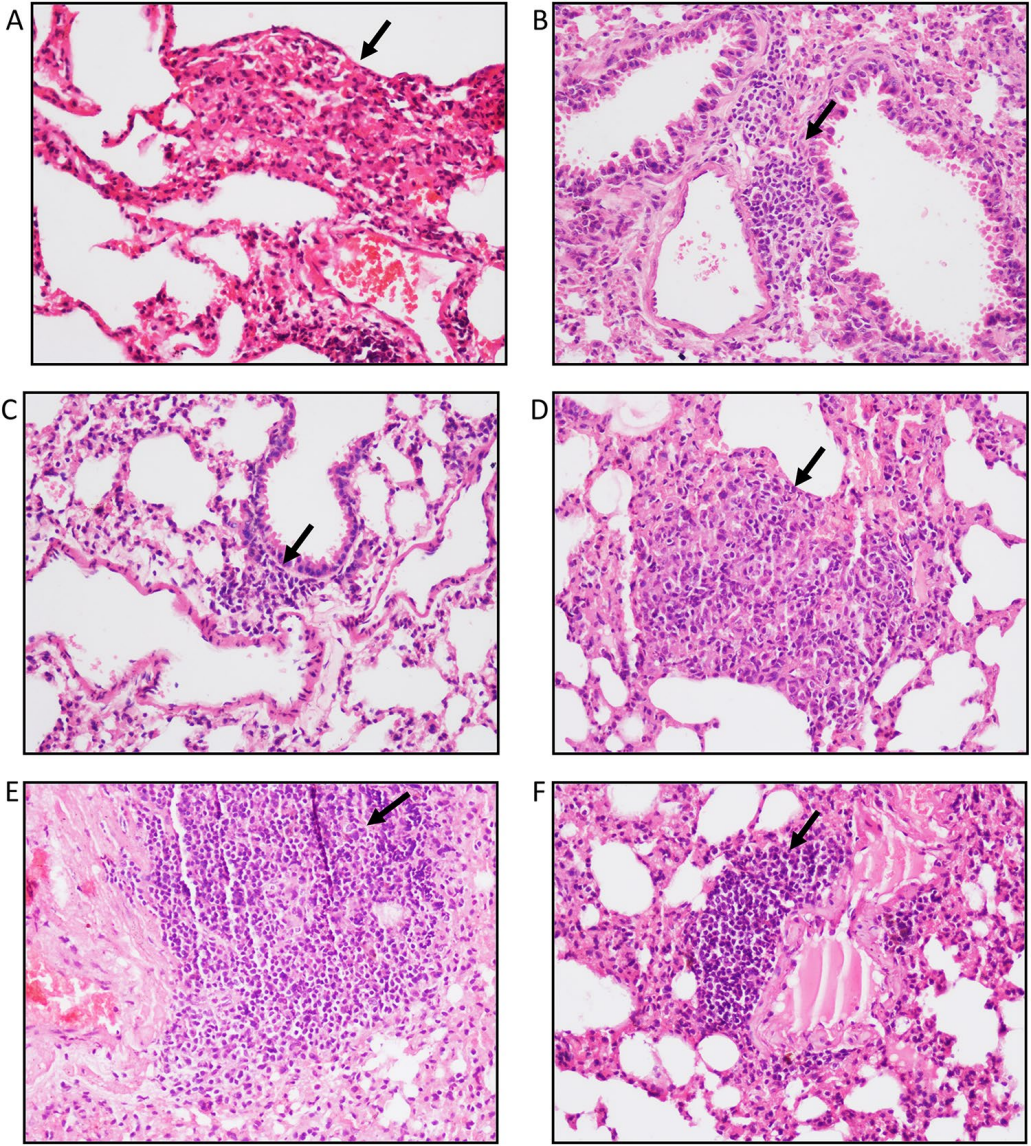
Histological examination of lung morphology to assess the presence of granulomas. After diabetes development, Hematoxylin & Eosin staining was performed on lung tissue of mice from Group-I, II and III at week 9 and 13 of streptozotocin treatment. Lung section of (**A**) Group-I mice (**B**) Group-II mice (**C**) Group-III mice after 9 weeks; Lung section of (**D**) Group-I mice (**E**) Group-II mice (**F**) Group-III mice after 13 weeks of streptozotocin treatment. Arrows indicate granulomas (**A**,**D**) and inflammation (**B**,**C**,**E**,**F**). Images were taken at 20× magnification. Group-I: latent tuberculosis only, Group-II: latent tuberculosis with diabetes and Group-III: latent tuberculosis with immunosuppression.

At week 9, ill formed granulomas were observed as represented by collections of epithelioid histiocytes along with the inflammation and haemorrhage in the lung tissues of animals from latent tuberculosis only group (Fig. 2A). In the lung tissues of animals from latent tuberculosis with diabetes group, peri-vascular and peri-bronchiolar inflammation was observed in lungs as indicated by the infiltration of lymphocytes with no granulomas (Fig. 2B). Similarly, in the lung tissues of animals from latent tuberculosis with immunosuppression group mild peri-vascular and peri-bronchiolar inflammation was observed but no granulomas were identified (Fig. 2C). At week 13, a collection of pale histiocytes admixed with surrounding lymphocytes and increased inflammation was observed in the lung tissues of Group-I animals (Fig. 2D). However, the lungs of latently infected mice with diabetes showed very dense peri-vascular and peri-bronchial inflammation. Ruptured air spaces were also seen in the lung tissue sections (Fig. 2E). In the lung tissue of Group-III animals, lymphocytic infiltration was observed (Fig. 2F). The level of significance between Group-I, II and III for granuloma formation, peribronchiolitis and perivasculitis at week 9 and 13 is given in Table 1. However, no changes were found in the spleen in all groups at week 9 and 13 of diabetes induction.

**Table 1:** Comparative histological scoring of different study groups.

Histological features	Time points	Group-I (mean score ± SE)	Group-II (mean score ± SE)	Group-III (mean score ± SE)	Level of significance for different group comparisons		
					Group-I vs Group-II (p-value)	Group-I vs Group-III (p-value)	Group-II vs Group-III (p-value)
Granuloma formation	Week 9	3.33 ± 0.33	0.33 ± 0.33	0.00 ± 0.00	*0.05	*0.05	ns
	Week 13	4.66 ± 0.33	0.00 ± 0.00	0.00 ± 0.00	*0.05	*0.05	ns
Peribronchiolitis	Week 9	3.33 ± 0.33	1.33 ± 0.33	2.00 ± 0.57	*0.05	ns	ns
	Week 13	3.66 ± 0.33	4.33 ± 0.66	1.33 ± 0.33	ns	*0.05	*0.05
Perivasculitis	Week 9	2.66 ± 0.33	2.33 ± 0.33	1.66 ± 0.33	ns	ns	ns
	Week 13	3.33 ± 0.33	4.33 ± 0.33	1.33 ± 0.33	ns	*0.05	*0.05

Mean score was calculated from 3 animals in each group. The data was represented as Mean ± SE of 3 animals in each group and the statistical significance was determined by Mann–Whitney test. *p ≤ 0.05. ns-non-significant. Group-I: latent tuberculosis only, Group-II: latent tuberculosis with diabetes and Group-III: latent tuberculosis with immunosuppression.

### Reactivation of *M. tuberculosis* by resuscitation promoting factors

**Figure 3:**
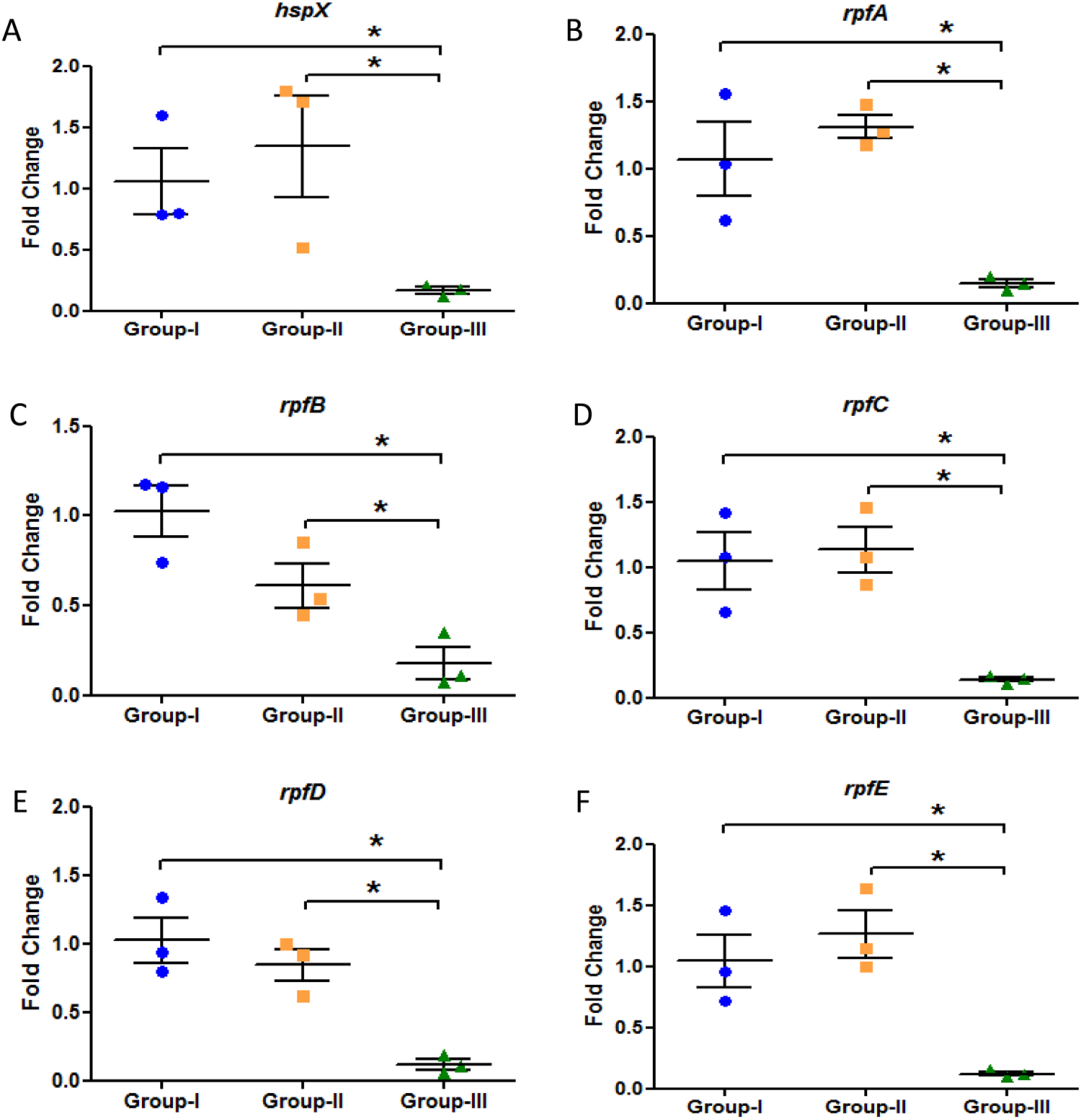
Expression analysis of various genes in the lungs of Group-I, Group-II and Group-III mice after 9 weeks of streptozotocin treatment. Gene expression of (**A**) *hspX* (**B**) *rpfA* (**C**) *rpfB* (**D**) *rpfC* (**E**) *rpfD* (**F**) *rpfE*. The data were represented as Mean ± SE of 3 animals in each group and the statistical significance was determined by Mann–Whitney test. *p ≤ 0.05. Group-I: latent tuberculosis only, Group-II: latent tuberculosis with diabetes and Group-III: latent tuberculosis with immunosuppression. Animal model was developed only once but gene expression was  studied in triplicates from each animal.

**Figure 4:**
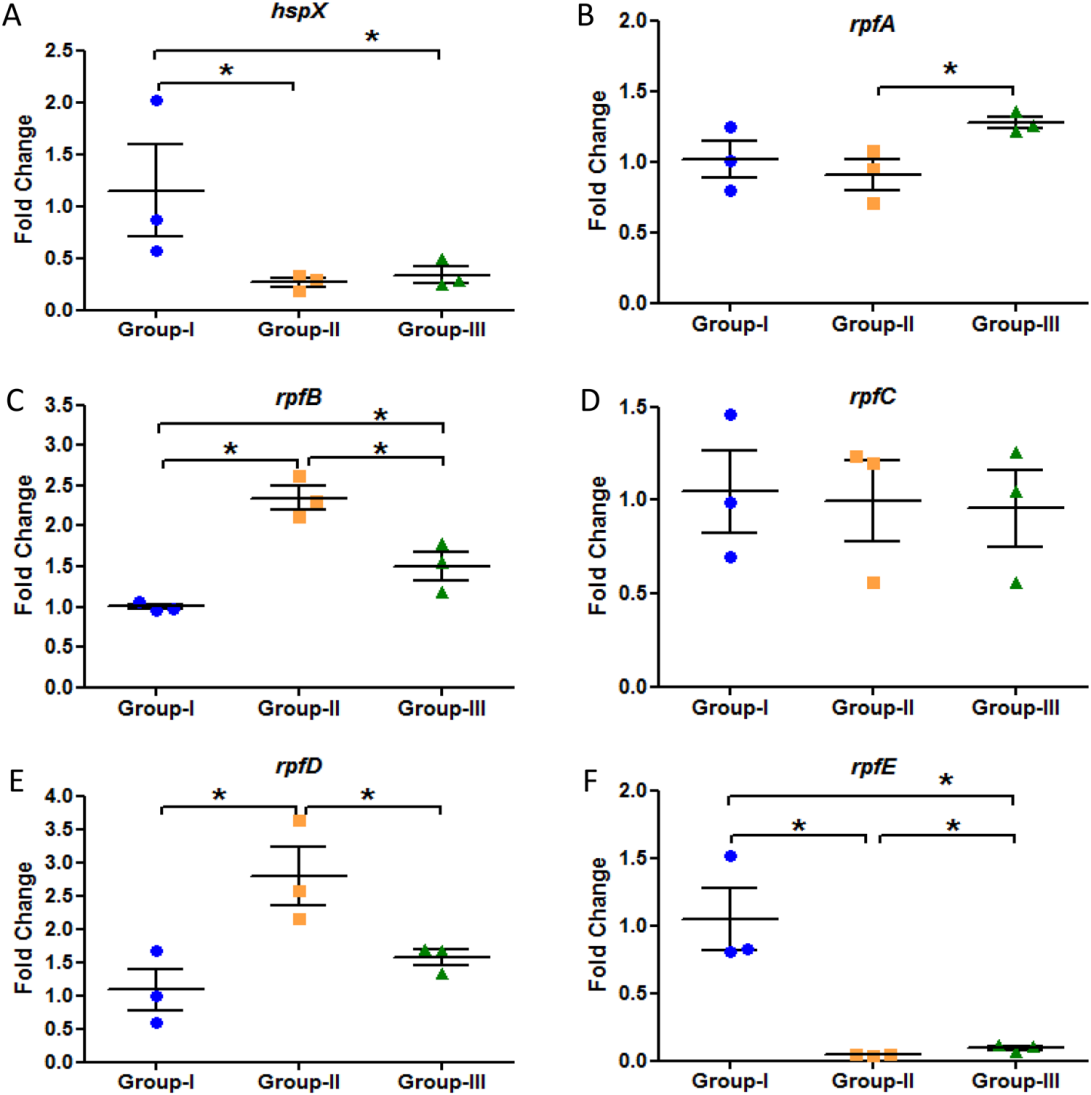
Expression analysis of various genes in the lungs of Group-I, Group-II and Group-III mice after 13 weeks of streptozotocin treatment. Gene expression of (**A**) *hspX* (**B**) *rpfA* (**C**) *rpfB* (**D**) *rpfC* (**E**) *rpfD* (**F**) *rpfE*. The data were represented as Mean ± SE of 3 animals in each group and the statistical significance was determined by Mann–Whitney test. *p ≤ 0.05. Group-I: latent tuberculosis only, Group-II: latent tuberculosis with diabetes and Group-III: latent tuberculosis with immunosuppression. Animal model was developed only once but gene expression was  studied in triplicates from each animal.

The expression of *hspX* gene was significantly downregulated in latent tuberculosis with immunosuppression group as compared to latent tuberculosis only group which signifies the activation from latency at week 9 (Fig. 3A). However, the expression of all the resuscitation promoting factor (*rpf*) genes was found to be significantly downregulated in latent tuberculosis with immunosuppression group (Group-III) as compared to latent tuberculosis only (Group-I) and latent tuberculosis with diabetes group (Group-II) at week 9 (Fig. 3B–F). Moreover, no significant change was observed in the expression of any of the genes mentioned above in latent tuberculosis with diabetes group as compared to latent tuberculosis only group after 2 weeks of diabetes development i.e. week 9, indicating that Group-II is behaving similar to Group-I (Fig. 3A–F). However, at week 13, a significant decrease was observed in the expression of *hspX* gene in latent tuberculosis with diabetes group (Group-II) as well as in latent tuberculosis with immunosuppression group (Group-III) as compared to latent tuberculosis only group (Group-I). No significant difference was observed in the expression of *hspX* between Group-II and Group-III (Fig. 4A). A significant increase was observed in the expression of *rpfB* gene i.e. approximately 2.3-fold, and *rpfD* gene, i.e. 2.8-fold in latently infected animals having diabetes as compared to animals having latent tuberculosis only (Fig. 4C,E). In latent tuberculosis with immunosuppression group also, around 1.5-fold increase was observed in the expression of *rpfB* gene as compared to latent tuberculosis only group but the expression was significantly decreased when compared to Group-II (Fig. 4C). The expression of *rpfA* gene was found to be upregulated in Group-III as compared to Group-II and the expression of *rpfC* gene remained same in all the groups whereas the gene expression of *rpfE* was found to be significantly downregulated in both Group-II and Group-III as compared to Group-I (Fig. 4B,D,F). It was observed that at earlier time point (week 9) Group-II behaved like Group-I but with increase in time duration (week 13), Group-II behaved similar to Group-III which indicates that the duration of hyperglycemia plays a vital role in modulating the expression of different resuscitation and dormancy associated genes. These findings suggest that diabetes promotes resuscitation of latent bacilli through *rpfB* and *rpfD* and thus favours its activation.

### Diabetes had no effect on treatment outcome

**Figure 5:**
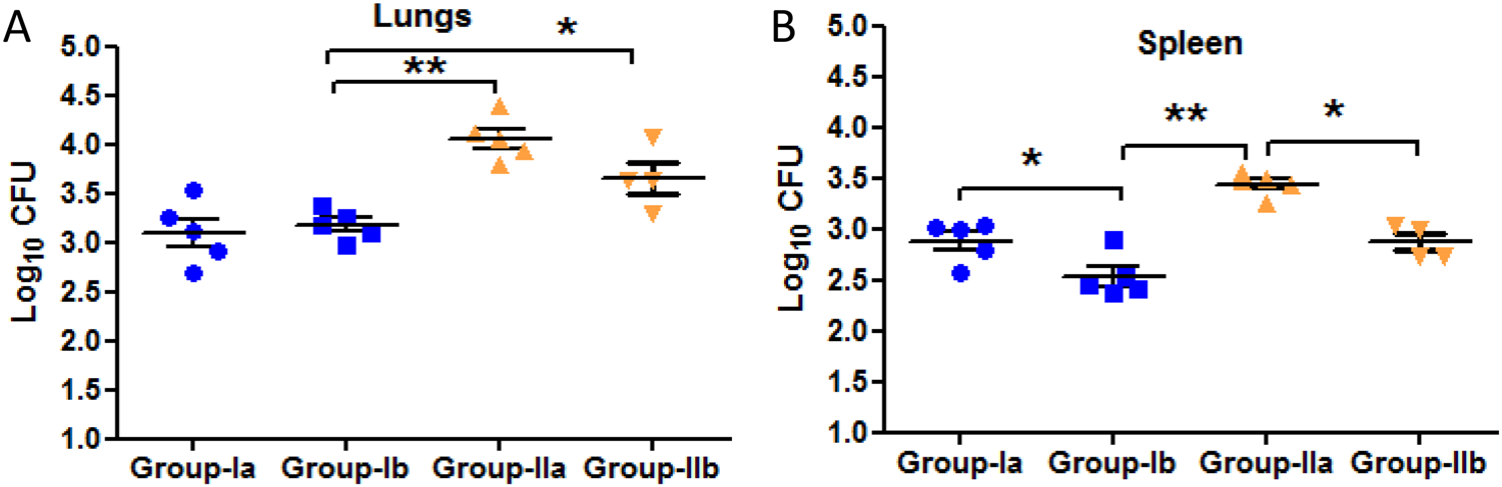
CFU comparison between untreated and treated groups. Log_10_ CFU counts in lungs and spleen of untreated and INH + RIF treated latent tuberculosis and latent tuberculosis with diabetes group (**A**) Lungs (**B**) Spleen. The data were represented as Mean ± SE of 5 animals in each group and the statistical significance was determined by Mann–Whitney test. *p ≤ 0.05. Group-Ia: latent tuberculosis untreated, Group-Ib: latent tuberculosis treated with INH + RIF, Group-IIa: latent tuberculosis with diabetes untreated, Group-IIb: latent tuberculosis with diabetes treated with INH + RIF. Animal model was developed only once but CFU enumeration was done in triplicates from each animal.

**Figure 6:**
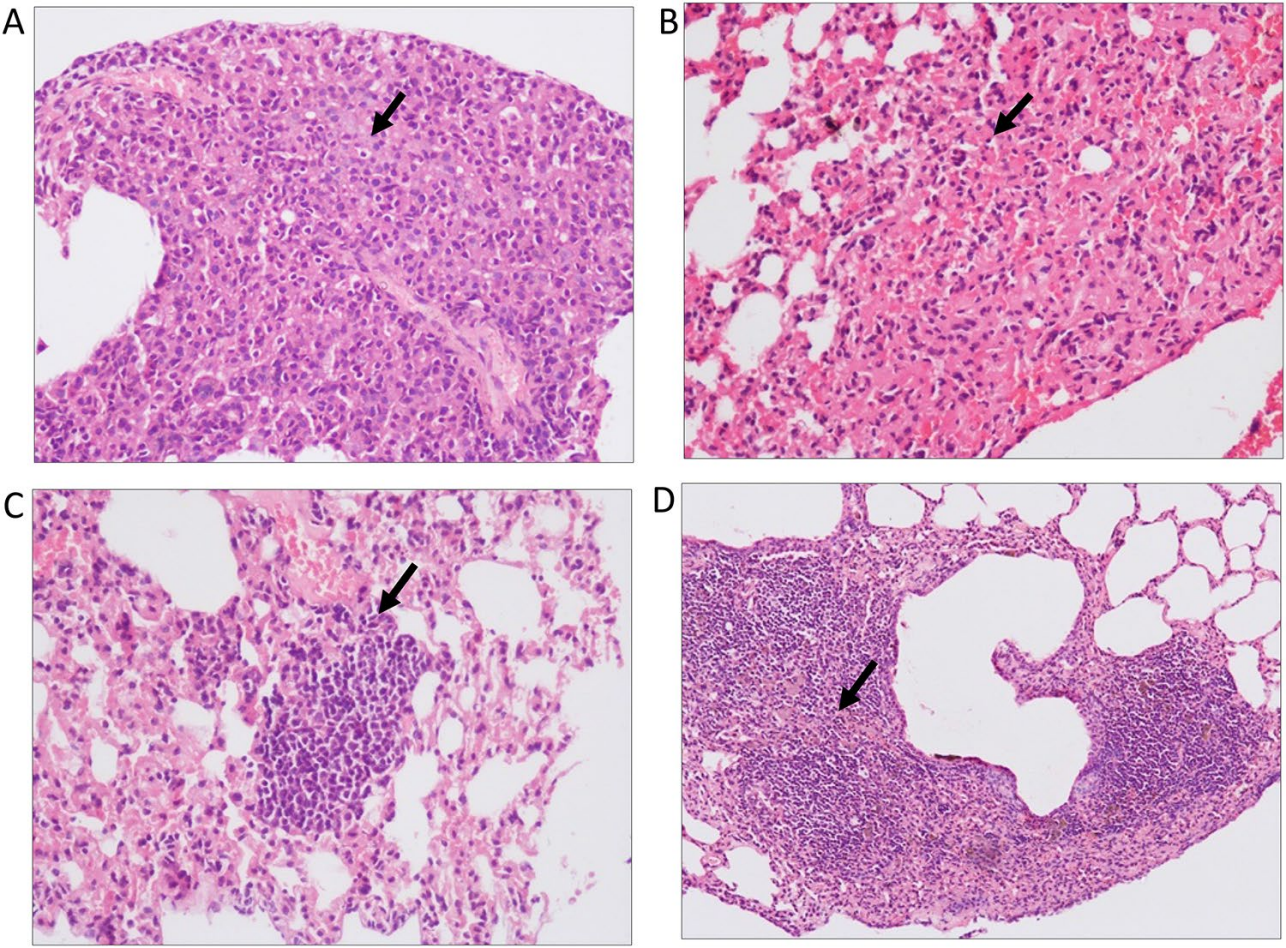
Histological examination of lung morphology to assess the presence of granulomas in untreated and treated groups. Hematoxylin & Eosin staining was performed on lung tissue sections of untreated and INH + RIF treated latent tuberculosis and latent tuberculosis with diabetes group. Lung section of (**A**) Group-Ia animals (**B**) Group-Ib animals (**C**) Group-IIa animals (**D**) Group-IIb animals after 4 weeks of treatment. Arrows indicate granulomas in lung sections (**A**,**B**,**D**) and inflammation (**C**). Images were taken at 20× magnification Group-Ia: latent tuberculosis untreated, Group-Ib: latent tuberculosis treated with INH + RIF, Group-IIa: latent tuberculosis with diabetes untreated, Group-IIb: latent tuberculosis with diabetes treated with INH + RIF.

**Figure 7:**
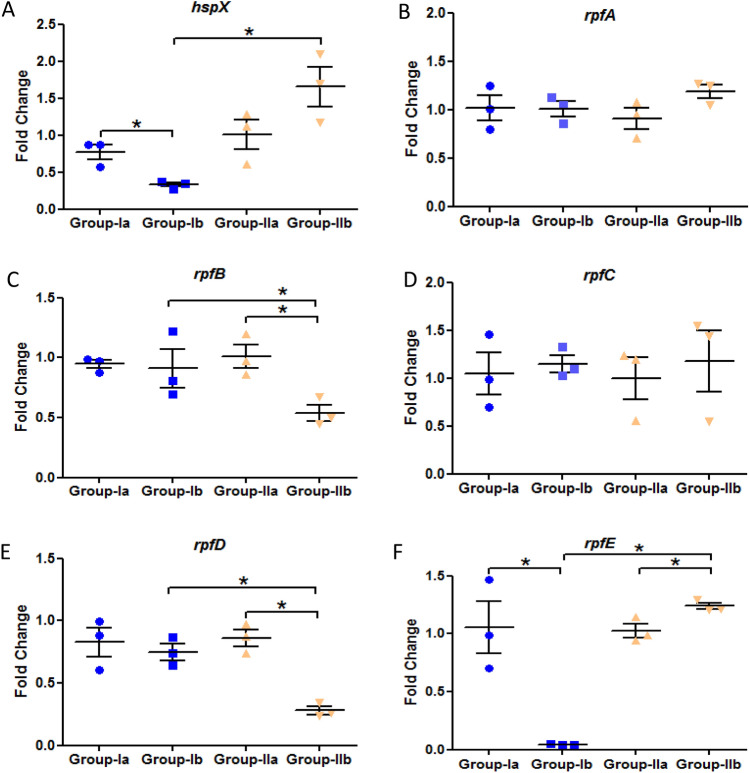
Gene expression analysis of various genes in untreated and treated groups. Gene expression of (**A**) *hspX* (**B**) *rpfA* (**C**) *rpfB* (**D**) *rpfC* (**E**) *rpfD* (**F**) *rpfE* in lungs of untreated and INH + RIF treated latent tuberculosis and latent tuberculosis with diabetes group. The data were represented as Mean ± SE of 3 animals in each group and no statistically significant changes were observed in the expression of hspX and rpf genes. Group-Ia: latent tuberculosis untreated, Group-Ib: latent tuberculosis treated with INH + RIF, Group-IIa: latent tuberculosis with diabetes untreated, Group-IIb: latent tuberculosis with diabetes treated with INH + RIF. Animal model was developed only once but gene expression was  studied in triplicates from each animal.

INH + RIF therapy had no effect on the bacillary loads in lungs of latently infected animals with or without diabetes. Although a significant decrease in log_10_ CFU/mL in spleen was observed after therapy in both latent tuberculosis only treated with INH + RIF (Group-Ib) and latent tuberculosis with diabetes treated with INH + RIF (Group-IIb) as compared to latent tuberculosis only, untreated (Group-Ia) and latent tuberculosis with diabetes, untreated (Group-IIa) respectively (Fig. 5A,B). After 4 weeks of treatment, granulomas were observed in lung tissues of Group-Ia (Mean score-4.66) and Group-Ib animals (Mean score-1.33) (Fig. 6A,B). In Group-IIa inflammation (Mean score-4.33) was observed but no granulomas were seen whereas in Group-IIb, histiocytic collection was seen and granuloma formation was observed (Mean score-0.33) (Fig. 6C,D). However, the changes were non-significant between untreated and treated groups. Moreover, a significant decrease in the expression of *hspX* gene was observed in Group-Ib as compared to Group-Ia but no significant change was observed in the expression between Group-IIa and Group-IIb (Fig. 7A). The expression of *rpfB* and *rpfD* was found to be significantly downregulated in Group-IIb as compared to Group-IIa but no change was observed in its expression between Group-Ia and Ib (Fig. 7E). The expression of *rpfE* was found to be significantly downregulated in latent tuberculosis treated group as compared to latent tuberculosis untreated group and expression was significantly upregulated in latent tuberculosis with diabetes treated group as compared to latent tuberculosis with diabetes untreated group (Fig. 7F). However, no significant changes were observed in the expression of *rpfA* and *rpfC* between untreated and treated groups (Fig. 7B,D). The results indicate that after treatment, observations of the latent tuberculosis with diabetes group (Group IIb) were not very different from only latent tuberculosis group. Thus, it was concluded that longer duration therapy i.e. more than 4 weeks is required to evaluate the treatment outcome.

## Discussion

Diabetes acts as a predisposing risk factor for tuberculosis making it necessary to delineate its role in LTBI activation. This prompted us to develop an animal model of latent TB with diabetes so as to thoroughly investigate the underlying factors that may lead to the reactivation of latent tuberculosis under diabetic conditions.

The conversion of LTBI to active TB was strongly supported by CFU counts which were found to be more than 10^4^. Moreover, a study by Dutta et al have shown similar results in which a model of latent tuberculosis was developed by immunization with rBCG and an increase in CFU counts was seen after treating the mice with anti-TNF-α mAb^18^. Similarly, increased CFU counts were seen after administration of aminoguanidine, a nitric oxide synthase inhibitor, resulting in immune suppression in latent tuberculosis mice model^19^. TNF-α neutralization has also been shown to reactivate chronic persistent tuberculosis in a low dose mice model of latent tuberculosis^20^. Abnormal regulation of inflammatory mechanisms in diabetes is also a characteristic feature that can cause exaggerated inflammatory responses in the lung, resulting in impaired lung function^21^. In the present study also, diabetes was found to be associated with compromised lung morphology with severe inflammation as indicated by lymphocytic infiltration and no granulomas. Our findings were supported by a study wherein granulomas became disorganised and inflammation was increased in the lungs of latently infected mice after treatment with anti-TNF-α mAb^18^. Another study on zebrafish infected with latent *M. marinum* also reported loss of granuloma integrity upon dexamethasone treatment^22^. Similar results were observed after blocking TNF-α in chronically infected C57BL/6 mice along with the dissolution of granulomatous tissues and increased lung inflammation^23^.

Alpha crystallin is believed to sustain the tubercle bacilli during latent or dormant phase of infection, thus indicating HspX as a potential marker of latent tuberculosis infection^24,25^. The gene expression of *hspX* was downregulated in latently infected mice induced with diabetes as compared to mice infected with latent tuberculosis only. This finding further confirmed the active state of tubercle bacilli after diabetes development. Increased expression of HspX in the latent state of tubercle bacilli and its reversal to normal levels during the exponential growth has also been reported earlier ^25^. Other studies also showed that the levels of anti-Acr IgG were lower in active TB as compared to latent TB thus supporting the present observation^26,27^. Similarly, a study by Muttucumaru et al strongly supported our findings and reported that alpha-crystallin was induced in the non-replicative state of *M. tuberculosis* as compared to active culture^28^.

Since TB reactivation has an inevitable link with resuscitation promoting factors, it was mandatory to study the expression of various *rpf* genes under diabetic conditions. The expression of *rpfB* and *rpfD* genes was significantly upregulated in latent tuberculosis and diabetes group in comparison to only latent tuberculosis group. These results strongly suggest that diabetes promotes resuscitation to latent tubercle bacilli and helps in its activation through resuscitation promoting factors B & D. Goldman et al have also shown the importance of Rpf in reactivation of chronic TB. RpfAB double knockout mutants exhibited a deficiency in reactivation in C57BL/6 mice upon immune suppression induced by nitric oxide synthase inhibitor (aminoguanidine) and by CD^4+^ T-cell depletion^29^. A delay in reactivation of bacterial growth upon immune suppression in the mouse model of chronic tuberculosis after deletion of *rpfB* gene has been shown^30^. Rpf triple and double mutants were observed to be defective in their ability to re-grow after immunosuppression induced by administration of aminoguanidine and anti-TNF-α antibodies^31^. A significant differential attenuation in virulence has been observed in the above stated triple mutants in a mouse model of chronic TB^32^. Kapoor et al reported that the dormant *M. tuberculosis* within the granuloma were resuscitated when treated with anti-TNFα-mAb resulting in increased expression of *rpf* genes i.e. *rpfA*, *rpfB* and *rpfC*^33^. Recently an Rpf-interacting protein A (RipA) was identified which interacts explicitly with RpfB and RpfE and efficiently promotes hydrolysis of peptidoglycan during reactivation and hence is responsible for the release of bacteria from dormancy^34^.

Since diabetes is associated with poor tuberculosis treatment outcomes, the effect of diabetes on LTBI treatment was studied. Latent TB therapy (INH + RIF) did not affect the bacillary load between untreated and treated LTBI with diabetes group. The results were supported by a clinical study wherein tuberculosis patients with diabetes showed poor TB treatment outcomes^35^. A meta-analysis study showed that tuberculosis treatment failure was higher in tuberculosis patients with diabetes^36^. In the present study, no significant difference was observed in the bacillary load between latent tuberculosis only untreated and treated groups, which could be because of the short treatment period. A study by Zhang et al showed that the therapy for LTBI (Rifapentine + Pyrazinamide and Rifapentine + Isoniazid + Pyrazinamide) was ineffective for less than 6–8 weeks^37^. Other studies have also shown that it takes 10–12 weeks for the standard regimen RIF + INH + PZA to cure active TB mice where the bacterial burden is high and 20–24 weeks to prevent relapse^38,39^. Thus, longer duration therapy (> 4–5 weeks) is required to eliminate minimal bacillary load. The gene expression of *hspX* was upregulated and the expression of *rpfB* and *rpfD* was downregulated in latent tuberculosis with diabetes treated group as compared to latent tuberculosis with diabetes untreated group. These results may suggest that upon treatment, *M. tuberculosis* may revert to the latent state which could be the reason for relapse of tuberculosis. However, it needs further exploration as therapy was given for only four weeks. Apart from the short duration of therapy, another limitation of the present study is that a single high dose of streptozotocin was administered to the animals to induce diabetes which is more reflective of type 1 diabetes and not of type 2 diabetes. The reason for using a high dose of streptozotocin is that animals were immunized with BCG which is thought to play a role in protection against diabetes. Several studies have shown that multiple low doses of streptozotocin did not induce diabetes in mice previously immunized with BCG^40,41^.

In conclusion, diabetes leads to disruption of granuloma formation and ultimately leads to the activation of latent tuberculosis. The major factors involved in this activation or resuscitation process are the resuscitation promoting factors *rpfB* and *rpfD* which can be targeted to contain the tuberculosis infection and reduce the shared burden of tuberculosis and diabetes co-pathology.

## Materials and methods

### Animal procurement and ethics statement

All animal procedures were performed in the Bio-Safety Level-III (BSL-III) facility at International Centre for Genetic Engineering and Biotechnology (ICGEB), New Delhi. Animal procedures were approved by the Institutional Animal Ethical Committee with ref No. 89/90/IAEC/616 and also by the Animal Ethical Committee of ICGEB, New Delhi with ref No. ICGEB/IAEC/02042019/TACF-PGIMER-16. All methods were performed in accordance with the relevant guidelines and regulations. Also, the study is reported in accordance with ARRIVE guidelines.

### Latent tuberculosis and diabetes in mice

**Figure 8:**
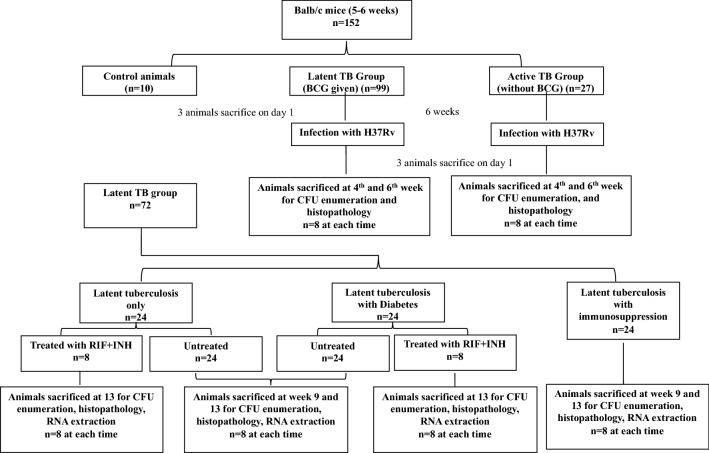
Flow chart depicting the experimental design, distribution of animals into different groups and time line of BCG infection, *M. tuberculosis* infection and subsequent treatments.

Latent tuberculosis mouse model was developed according to the method of Zhang et al. Briefly, BALB/c mice (5–6 weeks, either sex) were infected with BCG through aerosolization (O.D. of 0.5 at 600 nm ~ 5 × 10^6^ CFU/ml)^42^. After 6 weeks of immunization with BCG, animals were infected with *M. tuberculosis*  H37Rv (O.D. of 1.1 at 600 nm ~ 3.1 × 10^7^)^43^ through aerosol route. Animals were sacrificed after 4 and 6 weeks of infection for determination of latent TB development. Six weeks post infection with *M. tuberculosis* H37Rv, latently infected animals from latent TB group were divided in 3 groups i.e. Group-I: latent tuberculosis only, Group-II: latent tuberculosis with diabetes, Group-III: latent tuberculosis with immunosuppression. Group-II animals were induced with diabetes by using streptozotocin (150 mg/kg body weight) as described by Ventura-Sobrevilla et al.^44^. A flow chart of the distribution of animals into different groups with experimental endpoints is given in Fig. 8.

### Quantitative culture of lungs and spleen

Lungs and spleen were removed aseptically and homogenised in phosphate buffer saline (PBS). Aliquots (0.1 mL) of homogenates (undiluted and serial tenfold dilutions) were plated on Middlebrook 7H11 agar (Becton–Dickinson, USA) enriched with 10% oleic acid-albumin-dextrose-catalase (OADC) (Difco-Becton Dickinson, USA). 4 mg/ml of 2-thiophenecarboxylic acid hydrazide (TCH) (Sigma Aldrich) was added in 7H11 agar media to select for *M. tuberculosis*^45^. Plates were incubated at 37 °C in a 5% CO_2_ environment and colony forming unit (CFU) counts were determined after 28 days of incubation.

### Induction of immunosuppression in latently infected mice

Group-II animals were induced with diabetes and Group-III animals were treated with dexamethasone to induce immunosuppression after six weeks of infection with *M. tuberculosis* H37Rv. Group-III animals were used as a positive control for active tuberculosis. Three doses of dexamethasone was administered to Group-III animals at a dose of 6 mg/kg body weight subcutaneously 2 days apart^46^.

### Treatment therapy for latent tuberculosis

After the induction of diabetes, Group-I (latent tuberculosis only) and Group-II (latent tuberculosis with diabetes) animals were further subdivided into four sub-groups i.e. Group-Ia: latent tuberculosis untreated, Group-Ib: latent tuberculosis treated with isoniazid (INH) and rifampicin (RIF), Group-IIa: latent tuberculosis with diabetes, untreated, Group-IIb: latent tuberculosis with diabetes, treated with INH + RIF. 10 animals from both Group-I and Group-II each were treated with isoniazid (INH) and rifampicin (RIF). At week 9 of diabetes induction, Group-Ib and Group-IIb animals were administered with INH (10 mg/kg body weight) and RIF (10 mg/kg body weight) orally through gavage, 5 days per week for 4 weeks. After 4 weeks animals (n = 8) were sacrificed from all groups, their lungs and spleens were harvested and processed for CFU enumeration, histopathology and gene analysis.

### Histopathological studies

Aseptically removed lungs and spleen were transferred to 10% buffered formalin and further processed for paraffin embedding and sectioning^47^. Paraffin sections were deparaffinized and stained by standard haematoxylin and eosin (H&E) stain and acid-fast bacilli (AFB) stain. According to Valdez et al., a scoring system [0 = within normal limits (no change); 1 = minimal changes; 2 = mild changes; 3 = moderate changes; 4 = marked changes; 5 = very severe changes] involving examination of lungs for peribronchiolitis, perivasculitis and granuloma formation was used to give a total score for lungs from each mouse in all groups^48^.

### Gene expression analysis

Lung tissue was directly transferred to 15 ml of chilled 0.01% sodium dodecyl sulphate (SDS) and homogenized by using homogenizer. Homogenates were then centrifuged at 4000 rpm for 10 min at 4 °C to collect the bacterial pellets released from disrupted cells. The supernatant was separated and pellets were resuspended in 2–3 ml of TRIzol depending upon the size of the pellet. Samples were then transferred to screw cap tubes containing around 500 ml of 0.1 mm zirconia/silica beads and given 4 cycles of 30 s each with max. speed of Mini-beadbeater and processed for RNA isolation by standard phenol–chloroform method^49^. RNA (500 ng) was reverse transcribed using Revert Aid First Strand cDNA synthesis kit (Thermo Scientific, USA). qRT-PCR amplification was done by using SYBR^®^ Green chemistry on Roche LightCycler^®^ 96 Real-Time PCR Systems. The primer sequence used for the amplification of different genes is as follows:

- *rpfA*—GATGGACGCTCCGTTGGAC (F), GTTTGCTCGTTCACCGCAG (R)
- *rpfB*—TGGATGGTCACGACGCTAAG (F), CCAGATGCTTCCGTCGATCA (R)
- *rpfC*—GGTCGCGGTGCAATAGACA (F), TCAGCGCGGAATACTTGCC (R)
- *rpfD*—CTTACTACTGCGGGTGCTGG (F), CCCGAACAACCTCCAGTCTC (R)
- *rpfE*—TTGAAGAACGCCCGTACGAC (F), GTTCTCAGCCACCCGGATC (R)
- *hspX*—CACCACCCTTCCCGTTCAG (F), TGGACCGGATCTGAATGTGC (R)
- *16srRNA*—GTGGCGAACGGGTGAGTAAC (F), ATGCATCCCGTGGTCCTATC (R)

### Statistical analysis

The level of significance was determined by Mann-Whitney test and Kruskal–Wallis test using the GraphPad Prism, version 8.0 (GraphPad Software, San Diego, USA) (https://www.graphpad.com)
